# Pathologic Use of Video Games and Motivation: Can the Gaming Motivation Scale (GAMS) Predict Depression and Trait Anxiety?

**DOI:** 10.3390/ijerph16061008

**Published:** 2019-03-20

**Authors:** Sara Peracchia, Fabio Presaghi, Giuseppe Curcio

**Affiliations:** 1 Dipartimento di Medicina Clinica, Sanità Pubblica, Scienze della Vita e dell’Ambiente, Università degli Studi dell’Aquila, 67100 L’Aquila, Italy; sara.peracchia@gmail.com; 2 Dipartimento di Psicologia dei Processi di Sviluppo e Socializzazione, Sapienza Università di Roma, 00185 Roma, Italy; fabio.presaghi@uniroma1.it; 3 Dipartimento di Scienze Cliniche Applicate e Biotecnologiche, Università degli Studi dell’Aquila, 67100 L’Aquila, Italy

**Keywords:** motivation, videogaming, adolescents, adolescents, psychopathology

## Abstract

Videogaming is an increasingly prevalent activity among adolescents worldwide. The present study aimed at adapting the Gaming Motivation Scale (GAMS) to the Italian context, assessing its psychometric properties and verifying its sensitivity to predict depression and anxiety levels. From a sample of 1899 participants, a group of 388 adolescents who participated in the survey was divided into two subgroups of Heavy (HG, N = 188) and Light Gamers (LG, N = 200). A sub-sample of N = 172 adolescents also filled-in CESD and STAI to assess, respectively, depression and trait anxiety. Internal consistency and factorial structure of the Italian version of GAMS (GAMS-it) have been evaluated. Moreover, a latent regression structural equation model by predicting the CES-D and STAI scores with the GAMS-it factors has been carried out. GAMS-it has adequate validity and reliability levels, showing a very similar factorial structure to the original version. Therefore, this scale can be used to evaluate gaming motivation, which is useful for gaming motivation screening. Finally, it has been found that lower gaming motivation can be related to high level of depression and anxiety. The present findings provide a coherent picture, supporting the reliability and validity of the GAMS-it, that appears potentially useful in predicting anxiety and depression levels in a population of adolescents.

## 1. Introduction

In the last decade, a growing amount of literature has been proposed on the new phenomenon of excessive use of video games (VG), and one of the focal points of scientific debate is to clarify if videogaming can have the potential to modify users’ thoughts, feelings and behaviour. Looking at the results of previous investigations, conflicting opinions about the effects of exposure to VG did emerge: Some studies show the positive effects on some specific skills, as for example visual performance [1], visual acuity [2], probabilistic learning [3], stimulus-response mapping [4], encoding speed [5], and cognitive functioning [6]. On the other side, also some negative effects have been documented; as increasing aggression [7]; emergence of attention problems [8] or hyperactivity and mood troubles as depression and anxiety [9]; reduction of empathy [10]; impairment of social behaviour [11]; reduction of sleep time, quality and efficiency [12]; and possible addiction [13].

With respect to the issue of “addiction” to VG, researchers and clinicians have found considerable difficulties to assess, identify and define this phenomenon probably because VG does not involve a chemical substance and because problems induced by heavy use of these technological devices tend to be seen as “benign” since videogaming is less likely to pose social threats through illegal activities, as compared to drug addiction conditions [14]. However, research indicated that those who report excessive videogaming tend to show some addiction-like symptoms, including impairment in normal, social and occupational or educational functioning, tolerance, withdrawal and relapse [15], which may be considered “pathological” enough to require clinical attention and intervention. Only recently, the American Psychological Association included Internet Gaming Disorder (IGD) in the appendices of the DSM-5 [16], without specifically mentioning addiction to videogaming. Moreover, more recently, World Health Organization included the Gaming Disorder in the ICD-11 [17]. All these observations highlight the great relevance of these issues under both a scientific and health point of view.

Noteworthy, most studies payed attention only to the adult population, while this phenomenon is ever more relevant in adolescence. Converging evidences from different sources, in fact, indicate that adolescents’ use of VG is sharply increasing in recent times and that gambling is becoming a common activity among young people, particularly among boys [18], which is usually associated also to behavioral problems as for example, substance abuse [19]. It needs, in fact, to take into consideration that adolescence is a period of great risk for developing addictive behaviors: Following Johnston and colleagues [20], we know that before the age of 18 years, 60% of individuals start to use drugs and 80% to drink alcohol. A similar trend has also reported for cigarettes smoking (e.g., Reference [14]). Therefore, we cannot exclude the possibility that a long and continuous exposure to VG can become a real and structured addiction.

In the last decade, also in Italy, an increasing amount of young people has been found excessively involved in videogaming, an activity that might increase the possibility to develop addiction and/or gambling. To date, in Italy there are only a few instruments able to measure adolescent motivation to gaming, and a rapid and easy screening scale would be very useful. Such a kind of instrument could help in assessing motivation and involvement in videogaming in order to identify individuals “at risk”, and this could, in turn, help to prevent addiction and gambling [14].

In line with Self-Determination Theory (STD; [21]) it has recently developed the Gaming Motivation Scale (GAMS; [22]), designed to assess different aspects of gaming motivation: intrinsic motivation, integrated regulation, identified regulation, introjected regulation, external regulation, and amotivation. Motivation is one of principal components related to addiction e.g., Reference [23] and STD offers a multidimensional conceptualization of motivation that allows the assessment of level and type of motivation that can be applied to several domains including videogaming. Generally speaking, gaming motivation is a complex issue that includes different components: To the ones listed before and identified in the GAMS adaptation study [22] such as intrinsic and extrinsic motives for acting, we could include some others. For example, a sub-theory of SDT, called cognitive evaluation theory (CET [21]), is specifically focused on contextual factors that play a key role in intrinsic motivation. Such a theory hypothesizes that events and conditions able to boost an individual’s sense of autonomy and competence can sustain intrinsic motivation, whereas other factors that reduce these aspects tend to weaken this specific type of motivation; see Reference [24]. However, also other factors are associated with intrinsic motivation, such as presence (intended as the sense to be within the game world), intuitive control abilities (crucial to assess need satisfaction in game play), enhancement of individual well-being, and relatedness (when one feels connected with others). On the other hand, an increase of extrinsic motivation with its pivotal aspects of rewards, pressures and evaluations usually diminishes the weight of an intrinsic motive [25]. This complex interplay between different motivations and the several factors affecting them, can reasonably explain different aspects of gaming such as the need for satisfaction in games and short-term well-being; the appeal of violent game content; as well as motivational sources of post-play aggression; the antecedents and consequences of disturbed patterns of game engagement; or the causes and effects of games’ aspects as immersion, flow and presence, for a comprehensive review see Reference [25]. 

A companion issue to gaming is the psychological profile of gamers. As mentioned before, a relationship does exist between gaming abuse and some psychopathological traits such as anxiety and depression [9]. Some recent studies highlighted a reciprocal relationship between pathological gaming and indices of mental health troubles [26], indicating a kind of comorbidity of these traits with maladaptive gaming attitudes. Also, IGD was found to be strongly correlated with such psychopathological traits [27] while other authors hypothesized gaming as a possible maladaptive coping strategy to deal with negative affective disturbances [28].

The aim of the present study is to (a) adapt the Gaming Motivation Scale (GAMS; [22]) to the Italian adolescents’ context, (b) examine its psychometric properties in a sample of young Italian students, and (c) verify if GAMS-it factors might predict level of depression and trait anxiety. With respect to the last aim, we hypothesize that high gaming motivation positively correlates with gaming behavior and with psychopathological factors of anxiety and depression.

## 2. Materials and Methods

### 2.1. Participants

Different schools of Center-South Italy (I.I.S. “A. Bafile”, L’Aquila; I.S.I.S. “E. Mattei” Cerveteri [RM]; Liceo Classico “J. da Todi” Todi [PG]; Licei “T. Campanella”, Belvedere M.mo [CS]) have been invited to participate in the study, and a total of 1899 adolescents agreed to participate in the survey (970 males and 929 females; Mage = 15.13; SDage = 1.34 years; range 14–19 years). Together with the number of hours played, the whole sample was also asked to indicate the preferred type of VG. Participants chose different genres of VG (adventure, action, quiz, strategy, arcade, fighting game, role playing game, simulation, sports, educational, and first-person shooter). The different types of video games were divided into two main groups defined as “action video games” (AVG; games mainly based on action, incorporating adventure, action, strategy, fighting game, role playing game, simulation, sports, and first person shooter genres) and “no-action video games” (N-AVG; namely quiz, arcade, and educational kind of video games). Based on these data, it emerged that 40.65% of the total sample preferred AVG, compared with 4.58% that chose N-AVG.

Based on the amount of hours of videogaming, we selected 388 participants by the original sample who composed two subgroups of interest: 188 Heavy Gamers (HG), who reported to play for more than 4 h per week (145 males and 37 females; M-age = 15.12; SD-age = 1.42 years) and 200 Light Gamers (LG), who affirmed to play less than 1 hour per week (43 males and 163 females; M-age = 15.08 years; SD-age= 1.11 years). The analysis conducted on two subgroups highlights that for 70.59%, HG choose AVG, while LG prefer play more with N-AVG (61.34%). This sample of participants was considered for the confirmatory analysis of GAMS. 

Finally, among the 388 participants, a sub-sample of 172 participants (defined as Heavy Gamers N = 95, and Light Gamers N = 77) accepted to fill-in the CESD (Center for Epidemiologic Studies Depression Scale) and the STAI (State-Trait Anxiety Inventory) questionnaires. 

### 2.2. Instruments

In the present study, we administered to participants some different scales: (1) the Gaming Motivation Scale (GAMS) developed by Lafreniére and coworkers [22], (2) the Assessment of Internet and Computer Game Addiction Scale (AICA-S; [29]), (3) the Center for Epidemiologic Studies Depression Scale (CES-D; [30]), and 4) the State-Trait Anxiety Inventory (STAI; [31]).

The GAMS was generated on the basis of Deci & Ryan’s conceptual definitions of motivations [21]. The purpose of this scale was to investigate the reasons at the base of playing VG; participants were asked to respond to 18 items rated on a 7-point Likert scale (from 1 “do not agree at all”, to 7 “very strongly agree”) and bearing in mind a same basic question, i.e., “Why do you play to video games?”. The scale allows to assess different type of motivations: intrinsic motivation (desire to perform an activity for itself), integrated regulation (first aspect of extrinsic motivation, that refers to engaging in an activity out of choice), identified regulation (second aspect of extrinsic motivation, when people engage in a behavior based on its perceived meaning or its relation to personal goals), introjected regulation (third aspect of extrinsic motivation, that refers to the regulation of behavior through internal pressures such as anxiety and guilt, implying partial internalization), external regulation (fourth aspect of extrinsic motivation, that refers to behavior regulated through external means such as rewards), and amotivation (similar to learned helplessness, that refers to the relative absence of motivation either intrinsic or extrinsic) [22]. GAMS was translated into Italian by two experienced researchers; the translation was then evaluated by two independent experts in experimental psychology. Finally, one translator (an English native speaker) back-translated the questionnaire from Italian to English. After this procedure, the Italian version of the scale (GAMS-it) was obtained. For the general structure of the scale, we maintained the original one and thus the order of the items is the same as in the validation study (see Reference [22]).

The Assessment of Internet and Computer Game Addiction Scale (AICA-S; [29]) is a self-report scale for the assessment of potentially pathological computer and game behavior. Fifteen items are relevant for clinical classification of computer game use behaviour (e.g., craving, tolerance, loss of control, unsuccessful attempts to cut back, and withdrawal). Previous studies on its psychometric properties yielded satisfying results concerning item characteristics, reliability and validity [32]. For the present study, AICA-S was used for determining the amount of hours of gaming and to identify the participants defined as “Heavy Gamers” (HG) and “Light Gamers” (LG). In particular, we took into consideration the answer to the fourth question of this scale (“How you long are you playing computer games?”, with five response categories (1 = “less than one hour per week”, 2 = “1–2 h”, 3 = “2–4 h”, 4 = “4–6 h” and 5 = “more than 6 h to week”): participant who responded “1” were included in LG group, instead the participant that responded “4” and “5” were included in HG group. With respect to the meaning of question 1, “computer games” were intended as all the types of games both those on online platforms and on home console. The Italian version of AICA-S has been developed by following the same procedure followed with GAMS [33].

The 20-item Center for Epidemiologic Studies Depression (CES-D; [30]) is frequently used to estimate the prevalence of depressive symptomatology in the general population. Respondents rated the frequency with which they have experienced particular depressive symptoms during the past week. Answers to each item range from 0 (less than 1 day) to 3 (5–7 days) and are summed to compute a total score. It measures a single depression factor, ranging from 0 to 60; scores of 16 or above are considered potentially pathological.

The State-Trait Anxiety Inventory (STAI; [31]) is a commonly used measure of trait and/or state anxiety all over the world. It can be used in both clinical settings and research. The most popular version (Form Y) has 20 items for assessing trait anxiety and 20 for state anxiety: In the present study, we used the trait version. All items are rated on a 4-point scale (e.g., from “Almost Never” to “Almost Always”). Higher scores indicate greater anxiety level. 

### 2.3. Procedure

Participants were recruited in their school: Only those who were authorized by parents and teachers participated in the study; no incentive was given for participation. Participants completed the “paper and pencil” questionnaire in their class during lessons’ breaks under the supervision of at least one researcher, who was also available if participants asked for clarifications. 

The study is divided into two sections: The first one, preparatory to the latter, aimed at verifying the factorial structure of the GAMS-it and compared it with the original version; the second section was aimed at investigating its discriminant validity by correlating the six factors with psychopathological factors like those assessed by CES-D (depressive factors) and STAI (trait anxiety) and verifying if GAMS-it factors can predict the level of depression and trait anxiety.

The whole study protocol was conducted in accordance with the Declaration of Helsinki and approved by the Internal Review Board of the University of L’Aquila (#16/2016).

## 3. Results

### 3.1. Analysis Strategy

From a preliminary check for the normality of distribution, it resulted that all the 18 GAMS-it items reported a significant deviation from normality (Shapiro-Wilk test ranged from w = 0.45, *p* < 0.01, to w = 0.86, *p* < 0.01), so Robust Maximum Likelihood estimation method with robust standard error and robust fit statistics was considered for conducting the CFA (i.e., References [34,35]). The goodness of fit of the model was consequently evaluated by means of the Satorra-Bentler (SB) scaled chi-square statistic [34], as well as other well-known fit indices such as: Root Mean Square of Approximation (RMSEA; [36]), Comparative Fit Index (CFI; [37]) and Non-Normed Fit Index (NNFI; [38]). Following guidelines by Hu and coworkers (1992) [39], the model is considered to hold approximatively in the population if the RMSEA value is below 0.08 (the closer to 0.00 the better), and if both CFI and NNFI are above 0.95, it is indicative of a reasonable goodness of fit. The same fit indexes will be used to evaluate the fit for the latent factor regression model. 

Given that high- and low-frequency gamers (namely, HG and LG) may differ in terms of motivation and regulatory strategies, it is consequently crucial to test whether the same factor structure (in terms of latent means and covariance structure) is invariant across the two sub-groups. Multi-sample CFA was then performed in order to investigate how well the factor model emerging from the previous analysis could be generalized across high- vs. low-frequency gamers. 

A procedure for testing factorial invariance was followed (e.g., Reference [40]). The procedure consisted of a series of hierarchical statistical invariance tests (configural, metric, scalar, unique variance, latent variance and latent means), starting with the omnibus test of the equality of covariance matrices across groups. The scaled difference chi-square statistics, ΔSBχ^2^, [35] were used for comparing the fit between two nested models, i.e., configural and metric invariant and to determine if the more restricted model has or not a non-worsen fit than the less restricted model. The null hypothesis of the statistical test was accepted when the estimated probability of the test was greater than 0.01.

### 3.2. Results of CFA of GAMS-it

On the selected sample of participants who comprised of low (LG, N = 200) and high (HG N = 188) frequency gamers, a Confirmatory Factor Analysis has been performed. In general, the Six-factor structure reported satisfactory fit statistics (SB-χ^2^(120) = 256.35, *p* < 0.01) with an RMSEA of 0.070 (90% C.I: 0.058–0.082), a NNFI of 0.969, and CFI = 0.961. As shown in Table 1, all GAMS-it items reported a significant factor loading on the expected factor; moreover, as described in Table 2, all factors correlated significantly, and the expected direction and reliability was satisfactory (ranging from 0.76 to 0.93). It is worth noting, the correlation between Integrated regulation and Identified Regulation is extremely high (θ = 0.945) and it is of the same entity of that reported by Lafreniére and colleagues [22]. We tested the hypothesis that the correlation between the two factors is equal to 1, meaning that the two factors are the same thing. The difference in model fit was significant (ΔSB-χ^2^(1) = 12.23, *p* < 0.01) indicating that the model with the covariance fixed to 1 has a significantly worse fit. In conclusion, even if the correlation is high, the two constructs can be seen as different. 

### 3.3. Results of Multi-Sample CFA

Although the configural model had a significant SB-χ^2^, the values of RMSEA were acceptable while those of NNFI and CFI not (SB-χ^2^(240) = 743.33, *p* < 0.001, RMSEA = 0.05, NNFI = 0.86, CFI = 0.87). Under the assumption of strict metric invariance, the fit (SBχ^2^) did not worsen significantly (SB-χ^2^(252) = 779.91, *p* < 0.001, RMSEA = 0.05, NNFI = 0.88, CFI = 0.88; ΔSBχ^2^ (12) = 10.40, *p* = 0.58) so the same six factor model with the same loadings in the two groups is tenable. However when we add to the metric invariance also the invariance of intercepts, the model fits much worse (SB-χ^2^(264) = 896.72, *p* < 0.001, RMSEA = 0.05, NNFI = 0.84, CFI = 0.84; ΔSBχ^2^ (12) = 33.0, *p* = 0.0009). Given these results, we decided to do not proceed with the remaining hierarchical tests of measurement invariance, as the fit would surely worsen. In conclusion, the metric invariance for the two sub-groups was reached. 

### 3.4. Differences between Heavy Gamers and Light Gamers

Finally, we compared the Heavy (HG) and Light Gamers (LG) groups with respect to the six GAMS-it factors and to the CESD and STAI scores (Table 3). HG scored significantly higher than LG on Intrinsic Motivation, Integrated Regulation, Identified Regulation, Introjected Regulation, and on External Regulation, but not on Amotivation. Concerning psycho-pathological measures, we found that HG reported significantly lower scores than LG on STAI but not on CESD (Table 3). Nonetheless, it should be stressed that such a difference could be a direct consequence of unbalanced gender composition between HG and LG. 

### 3.5. Predicting Depression and Trait Anxiety with GAMS-it Factors

As stated before, the main objective of the study was to investigate discriminant validity aspects of GAMS by correlating the six factors of GAMS with psychopathological factors like those assessed by CES-D (depression level) and by STAI (trait anxiety). We performed a latent regression structural equation model by predicting the CES-D and STAI scores with the six latent GAMS-it factors and covarying for the effect of sex (Male vs. Female) and type of gamer (LG vs. HG). We preferred this statistical approach because it is a common model for two outcome variables, also giving the possibility of obtaining latent correlations. As for CFA and for MG-CFA, we used Robust estimation methods.

**Figure 1:**
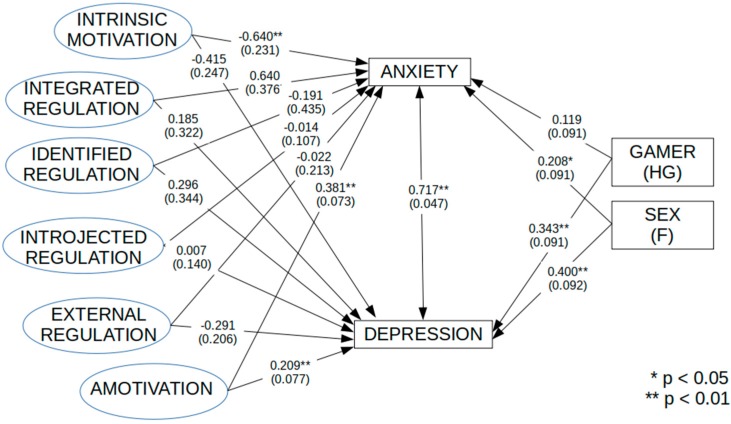
Path Model of the effects (completely standardized regression coefficients) of GAMS latent factors on Depression (CES-D scores) and Anxiety (STAI scores).

Results of the fitted Structural Equation Model (as depicted in Figure 1) showed the following fit indices: SB-χ^2^(180) = 484.94, *p* < 0.01, with a Robust RMSEA of 0.112 (90% C.I: 0.100–0.124), a NNFI of 0.857, and CFI = 0.888. Table 4 shows the estimated latent regression parameters of the six GAMS-it factors on both CES-D and STAI scores. Regarding CES-D scores, we found that only one of the six GAMS-it factors, Amotivation, significantly and positively predicts (b = 0.209, se = 0.077, *p* = 0.007) depression level. Females (b = 0.400, s.e. = 0.092, *p* = 0.000) as well as HG (b = 0.343, s.e. = 0.091, *p* < 0.001) reported a positive and significant effect. While concerning STAI scores, we found that not only Amotivation has a positive and significant effect (b = 0.381, se = 0.073, *p* < 0.001), but also Intrinsic motivation has a negative and significant effect (b = −0.640, se = 0.231, *p* = 0.006) on the trait anxiety measure. Also, in this case, females reported significantly higher levels of anxiety with respect to males (b = 0.208, s.e. = 0.091, *p* = 0.022); however, HG did not report anxiety scores significantly higher than that of LG (b = 0.119, s.e. = 0.091, *p* = 0.189).

Finally, it should be stated that the two clinical measures, CES-D and STAI, reported a significant and positive latent correlation (r = 0.717, se = 0.047, *p* < 0.001).

## 4. Discussion

In the present study, we firstly adapted the Gaming Motivation Scale (GAMS; [22]) to the Italian adolescents’ context, examining its psychometric properties in a sample of young Italian students and then assessed the ability of this instrument to predict the presence of possible psychopathological aspects in the young population.

To these aims, after having verified the factorial structure of the GAMS-it and compared it with the original version, we run a Confirmatory Factor Analysis on a subsample of 388 participants with low (LG) and high (HG) frequency of gaming. Results support the six-factor structure of GAMS-it reporting satisfactory statistics fit. As shown in Table 1, all GAMS-it items reported a significant factor loading on the expected factor and all factors correlated significantly and in the expected direction (Table 2); in addition, the reliability also resulted in being satisfactory. In addition, high correlation between Integrated Regulation and Identified Regulation (θ = 0.945) did emerge, as also reported in the original publication [22]. Moreover, the multi-sample CFA highlighted the equality between the various distributions of the correlation between the indicators and their dimensions, so the same six factor model with the same loadings in the two groups is tenable. The results confirmed the hypothesis of an invariant structure for the two groups. As a whole, such results showed adequate levels of validity and reliability of GAMS-it, as in the original validation study [22]. 

The comparison between two subgroups with respect to the six factors of GAMS-it and the result obtained from CESD and STAI showed a significantly higher score on Intrinsic motivation, Integrated regulation, Identified Regulation, Introjected Regulation, and External Regulation factor in HG, but not Amotivation. Moreover, HG showed a significantly lower score in the STAI, but not in the CESD (Table 3), indicating that a great amount of exposure to video games does not cause an increase of anxiety levels as usually believed. 

Another interesting result is the one emerging from Latent Regression Structural Equation Model correlating CESD and STAI scores with latent six factor of GAMS-it, showing satisfactory fit (SB-χ^2^(144) = 248.22, *p* < 0.01; Table 3). Regarding CES-D scores, only the GAMS-it’ Amotivation factor predicts significantly and positively the depression level (b = 0.247, se = 0.081, *p* < 0.001). This indicates that Amotivation levels can predict the depression level in a directly proportional way. Regarding STAI score, it emerged that not only Amotivation has a positive and significant effect (b = 0.390, se = 0.074, *p* < 0.001), but also that Intrinsic motivation has a negative and significant effect (b = −0.688, se = 0.238, *p* = 0.004). This indicates that Amotivation levels predict different levels of anxiety in a directly proportionate way; conversely, Intrinsic motivation levels predict indirectly the proportional anxiety levels. Finally, the two clinical measures, CES-D and STAI, reported a significant and positive latent correlation (r = 0.717, se = 0.047, *p* < 0.001). The present results only partially confirm the hypothesis of the study about a general correlation between motivation and anxiety/depression. 

All these results allow to make some interesting conclusions. From SEM results, three conclusions can be drawn. As a first, we can confirm that the original GAMS factor structure can be generalized to a different culture (i.e., the Italian one). This means that factors underlying motivations to play video-games are the same in the two cultures. Therefore, this scale can be used to evaluate gaming motivation, providing additional statistic support to GAMS and introducing in the Italian context a new scale to be used as a potential screening instrument of gaming motivation. Nonetheless, further research is mandatory in order to establish and clarify psychometric property of the GAMS-it. Secondarily, multi-sample SEM confirmed that the GAMS factor structure is invariant in Heavy and Light gamers; this does mean that motivations to play video-games in both populations did not differ in terms of types of basic drive and that the differences between HG and LG are limited to a quantitative difference, along the same factor structure: Heavy gamers show higher average values with respect to Light gamers on all GAMS dimensions, but one (i.e., Amotivation). Finally, consequent to this, the Amotivation factor is the only one that significantly predicts psychopathological traits like depression and anxiety. So the present study, in conclusion, shows (and adds to the literature) that motivation of both Heavy and Light gamers is the same, that it differs somewhat on average between the two groups, and that these differences may be used to discriminate between them; of greater importance, our study shows that Amotivation is not critical for differentiating HG from LG, but is crucial as a precursor of depression and anxiety, in the sense that high levels of Amotivation predict high levels of depression and anxiety scores, namely, lower gaming motivation can be related to high level of depression and anxiety. These data are in line with current literature on depression and anxiety symptoms in adolescence, where anhedonia, apathy and loss of interest for all kind of activity can predict psychopathological outcomes [41]. An opposite effect has instead been reported for Intrinsic Motivation factor (limitedly to STAI scale), showing that high levels of Intrinsic Motivation can predict a reduced level of anxiety.

The present data contribute to explain the not ever consistent literature on the effects of exposure to videogames on psychopathological components as depression and anxiety. For example, the relationship between videogaming and psychopathological symptoms is still unclear; is psychopathology to induce the approach to VG (and thus possible excessive use and addiction) or does the exposure to VG tend to unmask a latent condition of depression and anxiety? In our opinion, the results obtained in the present study can help to clarify the problem. Indeed, if a lack of gaming motivation predicts high levels of depression and anxiety, we could suppose that persons with depression and anxiety will not be interested in videogaming, as they are unmotivated. Therefore, a state of anxiety and depression cannot be considered a factor able to encourage the use of video games. This assertion is also confirmed by negative relationship between high Intrinsic Motivation to video games use and anxiety. Healthy participants seek wellness in video games, because technology increases well-being-inducing vigor and resistance in players [42].

Furthermore, research based on Self Determination Theory (SDT) revealed that self-determined forms of motivation induce adaptive consequences as pleasure, persistence and wellness [43]. This statement could also be extended to pathological gambling: Thus, people generally anxious and/or depressed would be not encouraged to play videogames or gambling because of their reduced levels of motivation. In this line, anxiogenic and depressive symptoms of patients with Internet Gaming Disorder (IGD) may emerge after exposure to video games. However, the multifactorial aspects of videogaming do not allow us to consider these definitive conclusions, and more research is needed to clarify the effective relationship among the different factors involved.

As a limitation of the study, the sample size should be highlighted. Further validation work should test the GAMS-it on larger samples in order to obtain a greater statistical validity. For example, a possible future direction could be a full cross-sectional sample, including also a group of “medium level” players. Moreover, another limitation arises from the limited availability of participants to complete psychopathological questionnaires. Also, the two subgroups (HG and LG) considered were unbalanced with respect to gender composition; this did not allow us to investigate the issue of gender weight, although it should be borne in mind that the differences between LG and HG with respect to psychopathological traits could arise by sample unbalancing. Finally, all data were collected using self-reports, which could lead to common problems of counterfeiting. Further studies are needed not only to replicate our results but also for testing the GAMS-it with multiple reports (i.e., relatives, friends) or with behavioral and objective measures.

Future prospects may be the application of GAMS-it on participants with IGD diagnosis, or to investigate the relationship between GAMS-it subscales, gender, and the preference for different kind of video games. Also, the correlation with other factors as aggression, impulsivity or addiction could be studied. Another issue to be studied is the correlation between videogames motivation and specific VG characteristics, namely graphic characteristics, immersivity, game option and history, and their potential link with mental and behavioral health [44]. Finally, it would be interesting to evaluate gaming motivation in different age ranges, in order to have an overview of gaming motivation at every time of life.

## 5. Conclusions

In conclusion, the present findings provide a coherent picture supporting the reliability and validity of the GAMS-it. Specifically, the results supported the internal consistency of the six subscales; the six-factor structure of the GAMS.it; interesting correlation with CES-D; and STAI scores.

The findings from the present study suggest that the GAMS-it is a valid assessment of gaming motivation in the Italian background and it significantly enables the prediction of anxiety and depression levels.

## Figures and Tables

**Table 1:** Standardized factor loadings with Robust SE for the six factors of GAMS-it (N = 388).

GAMS Factors and Related Items	Factor Loading	SE
Intrinsic motivation		
GAMS.1: Because it is stimulating to play	0.914 **	0.013
GAMS.2: For the pleasure of trying/experiencing new game options (e.g., classes, characters, teams, races, equipment)	0.857 **	0.022
GAMS.3: For the feeling of efficacy I experience when I play	0.881 **	0.015
Integrated regulation		
GAMS.4: Because it is an extension of me	0.896 **	0.020
GAMS.5: Because it is an integral part of my life	0.909 **	0.017
GAMS.6: Because it is aligned with my personal values	0.895 **	0.017
Identified Regulation		
GAMS.7: Because it is a good way to develop important aspects of myself	0.812 **	0.026
GAMS.8: Because it is a good way to develop social and intellectual abilities that are useful to me	0.726 **	0.036
GAMS.9: Because it has personal significance to me	0.844 **	0.022
Introjected Regulation		
GAMS.10: Because I feel that I must play regularly	0.894 **	0.020
GAMS.11: Because I must play to feel good about myself	0.837 **	0.028
GAMS.12: Because otherwise I would feel bad about myself	0.705 **	0.035
External Regulation		
GAMS.13: To acquire powerful and rare items (e.g., armors, weapons) and virtual currency (e.g., gold pieces, gems) or to unlock hidden/restricted elements of the game (e.g., new characters, equipment, maps)	0.850 **	0.025
GAMS.14: For the prestige of being a good player	0.907 **	0.016
GAMS.15: To gain in-game awards and trophies or character/avatar’s levels and experiences points	0.933 **	0.014
Amotivation		
GAMS.16: It is not clear anymore; I sometimes ask myself if it is good for me	0.924 **	0.032
GAMS.17: I used to have good reasons, but now I am asking myself if I should continue	0.761 **	0.037
GAMS.18: Honestly, I don’t know; I have the impression that I’m wasting my time	0.523 **	0.047

** *p* < 0.01; N = 388.

**Table 2:** Descriptive Statistics, Cronbach’ alphas and Correlations among the six GAMS-it factors (listwise missing deletion, N = 388).

	M	SD	α	Int-Mot	Int-Reg	Id-Reg	Introj-R	Ext-Reg	Amot
Int-Mot	3.18	2.03	0.91	1	0.823 **	0.867 **	0.780 **	0.853 **	0.268 **
Int-Reg	2.23	1.72	0.93		1	0.945 **	0.843 **	0.634 **	0.196 **
Id-Reg	2.22	1.56	0.84			1	0.816 **	0.710 **	0.205 **
Introj-R	1.90	1.45	0.85				1	0.706 **	0.248 **
Ext-Reg	2.86	2.14	0.92					1	0.254 **
Amot	2.51	1.70	0.76						1

Note: Int-Mot = Intrinsic motivation; Int-Reg = Integrated regulation; Id-Reg = Identified Regulation; Introj-R = Introjected Regulation; Ext-Reg = External Regulation; Amot = Amotivation [** *p* < 0.01].

**Table 3:** Descriptive statistics (M, SD) comparing Heavy Gamers (HG) and Light Gamers (LG) on each of the six GAMS-it factors and for CESD and STAI scores.

	HG			LG			F (1, 386)	*p*
	N	M	SD	N	M	SD		
Int-Mot	188	4.8	1.5	200	1.6	1.0	603.9	<0.001
Int-Reg	188	3.4	1.8	200	1.1	0.5	288.2	<0.001
Id-Reg	188	3.3	1.6	200	1.3	0.6	277.7	<0.001
Introj-R	188	2.8	1.6	200	1.1	0.4	202.0	<0.001
Ext-Reg	188	4.4	1.9	200	1.4	0.9	421.3	<0.001
Amot	188	2.6	1.6	200	2.4	1.7	1.2	0.283
CES-D	95	15.9	10.5	77	17.4	10.2	0.9 ^a^	0.349
STAI	95	39.8	10.4	77	43.6	11.2	5.3 ^a^	0.023

Note: Int-Mot = Intrinsic motivation; Int-Reg = Integrated regulation; Id-Reg = Identified Regulation; Introj-R = Introjected Regulation; Ext-Reg = External Regulation; Amot = Amotivation; CES-D = Center for Epidemiologic Studies Depression; STAI = State-Trait Anxiety Inventory. ^a^ Data for this combination of variables was available only on 172 participants. So d.f. for this ANOVA was (1, 170).

**Table 4:** Effects (Completely standardized regression coefficients) of GAMS latent factors on the CES-D and STAI scores (N = 172).

	CES-D Score			STAI Score		
	b	se	*p*	b	se	*p*
Int-Mot	−0.415	0.247	0.093	−0.640	0.231	0.006
Int-Reg	0.185	0.322	0.566	0.619	0.376	0.100
Id-Reg	0.296	0.344	0.389	−0.191	0.435	0.660
Introj-R	0.007	0.140	0.961	−0.014	0.107	0.893
Ext-Reg	−0.291	0.206	0.157	−0.022	0.213	0.919
Amot	0.209	0.077	0.007	0.381	0.073	0.000
Sex (F)	0.400	0.092	0.000	0.208	0.091	0.022
Gamer (HG)	0.343	0.091	0.000	0.119	0.091	0.189

Note: Int-Mot = Intrinsic motivation; Int-Reg = Integrated regulation; Id-Reg = Identified Regulation; Introj-R = Introjected Regulation; Ext-Reg = External Regulation; Amot = Amotivation.
